# The Effect of Short-Term Exposure to Lower Body Positive Pressure on Motor Signal Processing, Reaction Times, and Cardiovascular Parameters in Healthy Volunteers Using Medical Anti-shock Trousers

**DOI:** 10.7759/cureus.66266

**Published:** 2024-08-06

**Authors:** Keerthi Priya, Kishore K Deepak, Kanwal P Kochhar, Abhijith Anil, Dinu S Chandran, Shweta Sharma, Sunil Jangra, Ritesh Netam

**Affiliations:** 1 Department of Physiology, All India Institute of Medical Sciences, New Delhi, New Delhi, IND

**Keywords:** bereitshafts potentials, reaction time, medical anti shock trousers (mast), heart rate variability (hrv), lower body positive pressure (lbpp), microgravity

## Abstract

Microgravity, as experienced during spaceflight has notable effects on the cognition and cardiovascular systems. However, its effect on motor signal processing is not known. In this study, we planned to study the effect of microgravity simulation with a lower body positive pressure of 50 mmHg on motor signal processing, reaction times, and cardiovascular parameters. Thirty healthy human volunteers participated in this investigation, and continuous ECG and non-invasive blood pressure were measured at baseline, during, and after a lower body positive pressure of 50 mmHg. Bereitschafts potential was recorded at 0 mmHg and 50 mmHg pressure values in a lower body positive pressure (LBPP) suit. Parameters recorded during the pressure change of 0 mmHg to 50 mmHg were RR interval, heart rate, systolic blood pressure, diastolic blood pressure, stroke volume, cardiac output, and peripheral vascular resistance. Heart rate variability (HRV) was calculated from RR intervals during resting and pressure of 50 mm of Hg. We also compared simple and choice reaction times for visual and auditory stimuli during 50 mmHg LBPP exposure with baseline recording. We found a significant increase in systolic blood pressure, stroke volume, and cardiac output from baseline at 50 mmHg of LBPP. We found a significant change in amplitude and area of Bereitschaft potential at the C4 site at 50 mmHg of LBPP. We found a significant change in low-frequency power (LF) as compared to the baseline in HRV. Simple reaction time (visual & auditory) and auditory choice reaction time were improved at 50 mmHg of LBPP. Motor signal processing and reaction time were improved during 50 mmHg of lower body positive pressure exposure.

## Introduction

Human physiological systems have evolved over thousands of years to work in an upright posture on the Earth against downward gravitational force [1]. The manned missions to the moon and the upcoming space programs to Mars, where astronauts will spend years in space, demonstrate how humans have started to explore space extensively in the previous several decades. Currently, most space missions span several months in space, requiring astronauts' bodies to acclimate to weightless circumstances, particularly the cardiovascular system. In space, loss of gravitational pull causes the redistribution of body fluid [2]. Preparation for a space mission begins much before travelling to space so that the human body can adjust to the microgravity environment. Studying physiological adaptation to space and countermeasures to the microgravity situation on Earth is expensive and impractical. To overcome these problems, many Earth-based human microgravity experimental models are used on Earth to study the effect of microgravity on space [3]. Microgravity simulation methods in humans on Earth include dry immersion, head-down tilt bed rest (HDTBR), parabolic flight, unilateral limb suspension, and lower body positive pressure (LBPP) [4]. The LBPP chamber and suit (anti-gravity suit (G suit) and medical anti-shock trousers (MAST) are utilized to provide lower body positive pressure to simulate microgravity on Earth [5,6].

Microgravity simulation studies of acute and chronic exposure to LBPP reveal that at different pressures (10-50 mmHg), LBPP leads to significant changes in blood pressure, heart rate, forearm blood flow, forearm vascular resistance, and fluid redistribution in humans [7,8]. Similarly, microgravity simulation studies of HDTBR have shown that different degrees of head-down bed rest lead to significant changes in heart rate, blood pressure (BP), cardiac output (CO), and stroke volume. These findings are consistent with prior findings of chronic blood pressure, ECG, heart rate variability (HRV), and fluid redistribution in astronauts in space experiments [9-12]. Fluid redistribution in space and experimentally by HDTBR or LBPP exposure in labs led experts to propose that this could also affect cognition by influencing cerebral blood flow. Studies have well established that an increase in cerebral blood flow correlates with higher cognitive performance on an experimental task [13]. However, the effect of microgravity exposure in space experiments in astronauts and simulated microgravity experiments on Earth has revealed inconsistent results [14-16]. Therefore, this study was designed to investigate the acute effect of simulated microgravity on the executive function of healthy volunteers by comparing reaction times before and after exposure to 50 mmHg LBPP. Even though studies have been done on the effect of microgravity on cognition, the underlying process behind the change in the cognitive process has not been studied well. Only a few studies have used EEG, Functional near-infrared spectroscopy (FNIRS), and imaging tools to investigate the cognitive process behind the change in cognitive performance under simulated microgravity conditions [17]. These techniques explain changes in neurocognitive processes or increases in neuronal demand by measuring blood flow, but the other component of executive function is motor signal processing, which none of the previous studies have ever done. Therefore, this study was planned to investigate the change in motor signal processing upon exposure to 50 mmHg of LBPP.

This was the first study to investigate the effect of simulated microgravity on motor signal processing and executive function with cardiovascular parameter together in humans. We hypothesized that LBPP-induced simulated microgravity will impact cardiovascular parameters, hence affecting motor signal processing and executive function in participants.

## Materials and methods

The current study was a cross-sectional study. The study was approved by the Institute Ethics Committee for Postgraduate Research, All India Institute of Medical Sciences, with the approval number AIIMSA00435/13.12.2023. The study was conducted in the Neurophysiology Lab and Autonomic Function Test Lab at the Department of Physiology, All India Institute of Medical Sciences, New Delhi. After satisfying the inclusion (age group: 20-40 years, BMI: 18-25 kg/m^2^) and exclusion criteria (history of peripheral vascular diseases, cardiovascular disorders, and medications likely to affect autonomic tone), 30 healthy volunteers (age 22 ± 6 years) were recruited for the study. The subjects were informed about the nature and purpose of the study and the expected duration of the tests. The subjects were also informed about the confidentiality of their records. An informed consent was obtained from all the subjects' prior inclusion in the study.

**Figure 1:**
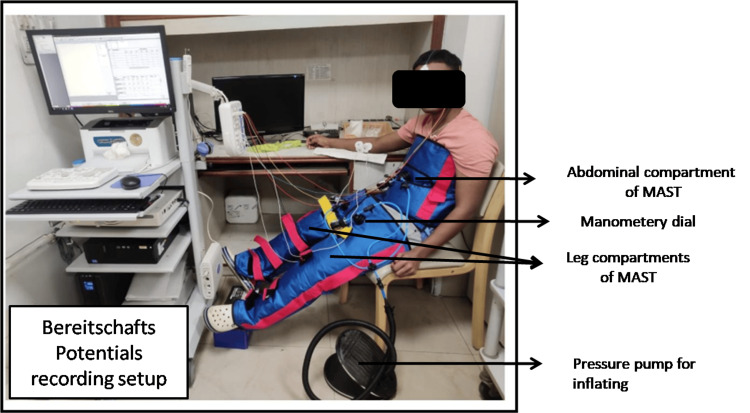
Bereitshafts Potentials recording wearing MAST The subject is performing Bereitshafts Potentials recording after wearing a medical anti-shock trouser (MAST).

Medical anti-shock trousers (MAST) are double-layered suits made of hard crepe cloth. These have three compartments, one tied around the abdomen and two side openings for the lower limbs. All three compartments are connected to a pressure pump for inflating and a manometer dial of 0-100 mmHg (Figure 1).

Protocol: The subject was asked to put on the antishock trousers without pressure, and then he/she was made to acclimate to the environment for 10 minutes. Initially, to see the effect of LBPP on cardiovascular parameters, we recorded blood pressure and heart rate throughout the procedure on the Lead II ECG. After a baseline recording of 10 minutes, LBPP was slowly increased to 50 mm Hg (Pr 50) in 10 min and maintained for 7 min, the pressure was released in anti-shock trousers, and the post-baseline recording was taken.

Cardio-vascular parameter recording: Non-invasive beat-to-beat blood pressure signals were obtained with the computer-based digital data collecting system Finometer® model 2 (Finapress Medical Systems, Amsterdam, Netherlands). The system consists of a main unit box and a front-end box with a finger cuff and a height adjustment unit attached. Signal acquisition was carried out utilizing preloaded software on the computer. Blood pressure signals were obtained using Penaz's volume clamp method. The sampling rate for acquisition was 1000 Hz, with a ceiling of 100 mmHg/V. Data collected were saved in computer memory for further offline analysis.

ECG was recorded throughout the process using a biopotential amplifier (ISOA-3) from Nevrokard, Slovenia, which was connected to the Power Lab TM System (AD Instruments, Australia). It included a 50-Hz notch filter to reduce noise from influences, as well as a 0.5-35 Hz band-pass filter. It has a highly efficient gain system. A three-pin ECG lead was used to record the ECG signal. The Lead II arrangement included shielded cables and disposable electrodes. The RRInterval will be calculated from the ECG during the trial.

Bereitshafts potentials recording: Bereitshafts potentials are motor-related cortical potentials (MRCP), which occur before actual movements and include planning and execution. Bereitshafts potentials are divided into two components. The early phase is the first negative slope that is associated with movement planning. The late phase consists of a rapidly increasing second negative slope that is associated with movement execution. For recording Bereitshafts potential, first, the subject was comfortably seated in the armchair. The scalp was washed, and conductive jelly was put on Ag/AgCl surface electrodes, and then electrodes were placed in the scalp at Fz, Cz, Pz, C3, and C4 sites. Electrode impedance was kept at <20 kohm. Baseline EEG recording was done before the wrist extension protocol. Brisk, self-paced right wrist extensions (based on the protocol, Neuropack x1 MEB 2300K (Nihon Khoden Japan) muscles (Extensor carpi radialis) were selected for EMG recording every 5-10 sec. Pre-set 50 contractions (trials) were used with different pressures in the LBPP suit. Offline analysis of waveforms was done after artifact rejection [18].

Reaction time recording: The reaction time testing was done on Audio Visual Reaction Timer (RMS, Recorders and medicare system (P) LTD, Haryana, India). The Reaction time was recorded in all the volunteers in the morning (10-10:30 am). For simple visual reaction time, subjects were instructed to stop the switch as soon as they perceived the visual signal. For simple auditory reaction time, subjects were instructed to stop switching as soon as they perceived the auditory signal. For choice visual reaction time, subjects were instructed to respond differentially to two different colors of light singles as soon as they perceived the visual signals. For choice auditory reaction time, subjects were instructed to respond differentially to two different auditory signals as soon as they perceived the sound signals. Three day's training session was given to each subject before the test day. Subjects were instructed to get proper sleep of eight hours before the day of the test.

Data analysis

The averaging of five minutes of beat-to-beat blood pressure and RR interval recording was done at baseline, 50 mmHg of LBPP, and post-LBPP and the delta change in each systolic blood pressure (SBP) and diastolic blood pressure (DBP), mean blood pressure (MBP), and heart rate (HR) was computed at 50 mmHg from baseline. Heart rate variability was assessed from a beat-to-beat RR interval derived from Lead II ECG data. All recording procedures for HRV were in accordance with the recommendations of the European Task Force. The heart rate variability was calculated from the recorded ECG with the help of Lab Chart Pro 8® software (ADInstruments, Dunedin, New Zealand). Firstly, the RR intervals were computed from ECG by the ECG software module of Lab Chart Pro. The whole recording was divided into three sets for HRV analysis at baseline, 50 mm Hg of LBPP, and post. Time series analysis represents the means of evaluating variability and identifying measures of variation over time. Time series analysis parameters were NN intervals or instantaneous heart rate (SDNN) and differences between the NN intervals (SDSD, NN50, pNN50). Frequency domain analysis was done using the intervals between consecutive R waves as the tachogram, and the parameters included were very low-frequency range power (VLF), low-frequency range power (LF), high-frequency range power (HF), and the LF/HF ratio.

Statistical analysis 

All statistical analyses were performed using Graph Pad Prism (version 5.0) (Graph Pad Software, Inc., USA). Each parameter was tested for distribution based on standard normality tests (D Agostino-Pearson omnibus normality test, Kolmogorov-Smirnov, and Shapiro-Wilk test). Gaussian data were represented as mean ± SD and non-Gaussian distribution was represented as median (interquartile range). To compare two groups of data, Paired T-test and Wilcoxon signed rank were used according to the distribution of data. Comparison of cardiovascular parameters between different groups was done by ANOVA with repeated measures or Friedman test according to the distribution of data and post hoc test e.g., Tukey's multiple comparisons test and Dunnett's multiple comparisons test were used respectively. Level of significance were expressed as *P < 0.05, **P < 0.01, ***P < 0.001.

## Results

The subjects screened for the study were recruited only if they satisfied all the inclusion criteria and were excluded according to the exclusion criteria. A total of 30 healthy volunteers were recruited for the study after explaining the procedure, and then written informed consent was obtained. ECG-Lead II and Bereitschafts potential were recorded in all 30 subjects. The recorded data was evaluated for artifacts. The data for all 30 subjects was found to be free of movement artifacts and was analyzed.

Effect of microgravity on cardiovascular parameters

There was a significant increase in the systolic blood pressure during 50 mmHg of LBPP from baseline as observed, and there was no significant difference in the diastolic blood pressure as observed. There was no significant change in the heart rate during 50 mmHg of LBPP from baseline, as observed. Stroke volume, cardiac output, and peripheral resistance were derived from beat-to-beat blood pressure recording by using the Windcessel model. There was a significant change in stroke volume and cardiac output during 50 mmHg of LBPP from baseline. There was no significant change in peripheral vascular resistance at 50 mmHg of LBPP from baseline (Table 1).

**Table 1:** The effect of microgravity simulation using lower body positive pressure (LBPP) on various sites cardiovascular parameters and heart rate variability Total peripheral vascular resistance (TPVR), Standard deviation of differences between adjacent RR intervals (SDSD), The square root of the mean of the sum of the squares of the differences between adjacent RR intervals (RMSSD), and percent of the number of pairs of adjacent RR intervals differing by more than 50 ms in the entire recording ( pNN50)  (time domain analysis), power in the low-frequency range-0.04 to 0.15 Hz (LF), power in the high-frequency range- 0.15 to 0.4 Hz (HF), and total power of variance of all RR intervals (Total power) (frequency domain analysis). Data: N=30, Data represented as  Mean±SD or Median (IQ range). *P < 0.05, **P < 0.01, ***P < 0.001 Baseline vs Pr 50; # P < 0.05, # # P < 0.01, # # # P < 0.001 Baseline vs Post.

Parameters	Baseline	Pr 50	Post	P value
Systolic Blood pressure (mmHg)	111.2 ± 10.1	117.5 ± 11.7*	111.7 ± 13.0^#^	< 0.0001***
Diastolic blood pressure (mmHg)	67.40 (55.30-72.18)	69.13 (55.44 - 72.28)	69.13 (55.44 -72.28)	0.0129*
Heart rate	81 ±13	82±12	84 ±13	0.4357
Cardiac output	6.5 ± 1.009	7.0 ± 1.26*	6.6 ± 1.19	<0.0001***
Stroke volume	76 ± 9.3	84 ± 10.4*	78 ± 12.05	<0.0001***
TPVR	0.7 ± 0.11	0.6 ± 0.30	0.7 ± 0.11	0.2894
Parameters	Baseline	50 mm Hg	Post	p value
SDSD (ms)	31.10 (20.35-40.90)	32.90 (23.15-36.45)	35.20 (25.70-40.21)	0.5572
RMSSSD (ms)	33.80 (20.90-41.90)	33.90(24.20-37.00)	35.4 (27.00-45.90)	0.2111
Pnn50 (%)	3.70 (1.27-6.72)	3.65 (2.22-6.15)	4.25 (2.40-6.50)	0.789
LF (ms^2^)	173.2 (135-197.4)	237.4 (146-289.6) *	218 (142-260.4)	0.0404*
HF (ms^2^)	229.7(144-278.2)	239 (206.5-298.8)	241 (228.5-241.3)	0.8640
LF: HF	1.89 (1.36-2.60)	1.98 (1.25-2.87)	2.50 (1.40-4.30)	0.4224
Total Power (ms^2^)	1091 (948.7-1165)	1085 (984.2-1181)	1075 (1053-1125)	0.7739

Effect of microgravity on autonomic profile

Heart rate variability was recorded at baseline, at LBPP exposure (50 mm Hg), and post LBPP exposure. The lead II ECG signal was analyzed to assess heart rate variability in the time and frequency domains of cardiac autonomic function at baseline and Pr 50 mmHg of LBPP simulated on motor planning.

Time Domain: The overall heart rate variability analyzed using SDNN (standard deviation of the NN interval) showed no significant change in time domain parameters at 50 mm Hg of LBPP compared to baseline. The SDSD (standard deviation of differences between adjacent NN intervals) and pNN50 (NN50 count divided by the total number of all NN intervals) were calculated for estimation of the parasympathetic component of HRV; no significant change was found in time domain parameters for at 50 mmHg of LBPP in comparison to baseline.

Frequency domain: There was a significant increase in the low-frequency component and Lf/HF ratio of the frequency domain parameter at 50 mmHg of LBPP from baseline. No significant change was observed in HF (nu), VLF (nu), HF/LF, and total power in the frequency domain at 50 mmHg of LBPP in comparison to baseline (Table 1).

**Figure 2:**
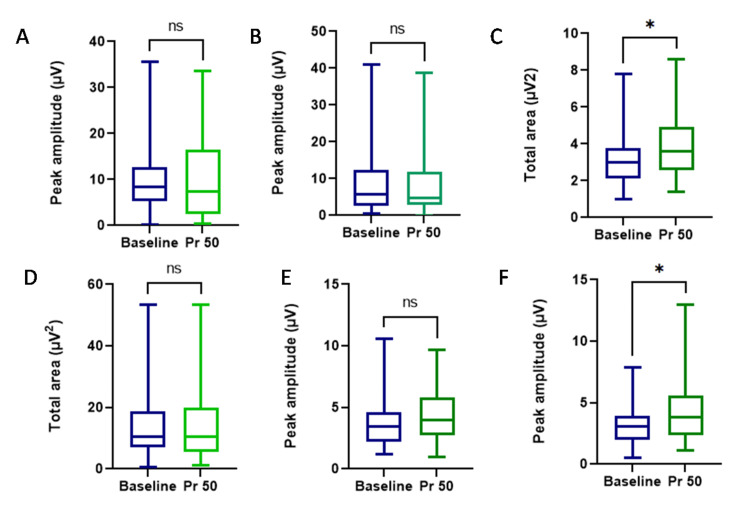
The effect of microgravity simulation using lower body positive pressure (LBPP) on various sites of Bereitschafts potentials: Peak amplitude on (A) Cz, (B) C3, (C) C4 Total area (D) Cz, (E) C3, (F) C4. N=30 Data are expressed as median (range). *P < 0.05, **P < 0.01, ***P < 0.001 compared with baseline by paired T test. We found a significant change in the peak amplitude (P= 0.0419) and total area (p= 0.0302) of C3 after LBPP exposure of pressure 50 mmHg.

The effect of microgravity simulation on various sites of Bereitschafts potentials: no significant changes in amplitude and area of Bereitschafts potential in the A1 site after LBPP exposure compared to baseline. There were no significant changes in amplitude Bereitschafts potential and area in the A2 site after LBPP exposure compared to baseline. There was a significant increase in the peak amplitude and area of BP of the A3 site post LBPP exposure compared to baseline (Table 2, Figure 2).

**Table 2:** The effect of microgravity simulation using lower body positive pressure (LBPP) on various sites of Bereitschafts potentials N=30, Data represented as  Median (IQ range). *P < 0.05, **P < 0.01, ***P < 0.001 Baseline vs Pr 50; # P < 0.05.

Peak amplitude (µV)	Baseline	Post Pr 50	P Value
Cz	5.75 (2.7-12.48)	4.7 (2.87-11.85)	0.903
C3	3.45 (2.175-4.62)	4 (2.77-5.77)	0.271
C4	2.1(1.4-3.5)	3.6 (2.55-4.93)	0.0419*
Total area (µV2)			
Cz	8.45 (5.3-12.68)	7.45 (2.4-16.45)	0.59
C3	10.6 (6.97-18.73)	10.55 (5.47-19.88)	0.654
C4	3.1 (2-3.9)	3.8 (2.3-5.6)	0.0302*

The effect of microgravity simulation on reaction time

**Figure 3:**
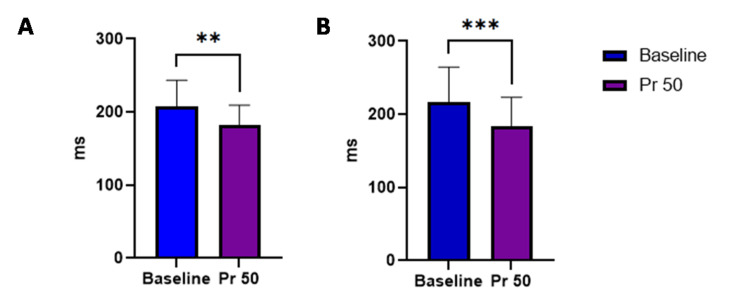
Effect of Pr 50 of LBPP on simple reaction time A) visual reaction B) auditory reaction time. N=30 Data are expressed as mean ± SD. *P < 0.05, **P < 0.01, ***P < 0.001 compared with baseline by Student’s t test. A significant change was seen in visual (** p= 0.0043 )and auditory reaction time (*** p=0.0009 ) during LBPP of pressure 50.

**Figure 4:**
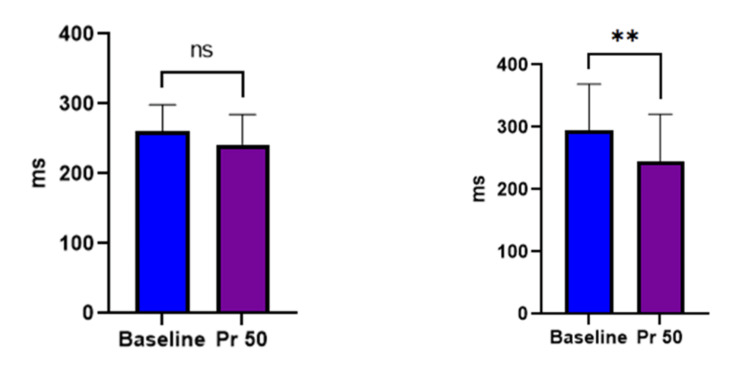
Effect of Pr 50 of LBPP on choice reaction time A) visual reaction B) auditory reaction time. N=30 Data are expressed as mean ± SD. *P < 0.05, **P < 0.01, ***P < 0.001 compared with baseline by Student’s t test. A significant change was seen in auditory reaction time during LBPP of pressure 50 (p= 0.0017).

To check the executive functions during exposure to simulated microgravity using LBPP, we assessed the simple and choice reaction time. We found that there was a significant change in simple visual (207 ± 36.16 vs 182± 27.33) p= 0.0043 and auditory reaction time (217±47.6 vs 183± 39.6) p=0.0009 on exposure to pressure 50 mmHg of LBPP. Similarly, we found a significant change in auditory choice reaction time (295.2±74.05 vs 244.4± 76.45, p=0.0017), but choice visual reaction time did not show any significant change (260.1±38.40 vs 241.9 ± 43.90, p=0.1036) (Figures 3, 4).

## Discussion

The study was conducted to investigate the effect of microgravity simulation on reaction time, motor signal processing, and autonomic profiles in humans. The study was conducted on 30 healthy volunteers who were subjected to 50 mmHg of LBPP for 7 minutes each. The study quantifies the effect of LBPP on readiness potential and heart rate variability.

In the present study, the beat-to-beat blood pressure was continuously measured at baseline, Pr 50, and post-LBPP. We recorded changes in the SBP, DBP, HR, stroke volume, CO, and peripheral vascular resistance (PVR). We found that after exposure to 50 mmHg pressure in LBPP, the systolic blood pressure tends to increase compared to the baseline of LBPP. Similar observations have been made earlier on the effects of posture on cardiovascular responses to lower body positive pressure at rest and during dynamic exercise with changes in the blood pressure with grades of LBPP. Similarly, other studies have shown an increase in cardiac stroke volume (SV) and CO at 25, 50, and 75 mmHg of LBPP [19], while others have shown a decrease and no effect of LBPP on CO at 30-mmHg LBPP [9]. This could be due to an increase in sympathetic activation due to LBPP. We did not find any change in PVR, but we did find a change in SV, which might be due to a change in sympathetic outflow, as we found a significant change in the LF power at 50 mmHg of LBPP. No change in PVR showed probably no change in venous return. But Nishiyashu et al. (2007) have shown an increase in PVR [20]. Sympathetic stimulation during 50 mmHg LBPP might be due to the activation of muscle mechanoreflex during lower body positive pressure [21] and a change in the transmural pressure of lower limb vessels, as shown by Reneman et al. (1980) [22].

In the present study, it was found that the heart rate did not change from baseline with 20 mmHg and 50 mmHg of LBPP. Nishiyashu et al. (1985) reported similar observations with no changes in heart rate at 25 and 50 mmHg in the supine position [19]., but Sucky et al. (2018) reported a decrease in 20-23% heart rate in anti-gravity treadmills facilitating locomotion by LBPP. Further analysis of heart rate variability was done to assess the role of the sympathetic component during 50 mm Hg of LBPP, simulating the effect of microgravity. No changes were observed in the time domain, but in the frequency domain, the low-frequency component increased with a decrease in the HF component, thereby decreasing the LF:HF ratio at 20 mmHg of LBPP from baseline. Similar results have been reported with studies on the effect of LBPP on cardiorespiratory at rest and during sub-maximum exercise; however, no significant change in the other frequency domain of heart rate variability was found at 50 mmHg of LBPP from baseline. In the present study, a significant increase in sympathetic activity (low-frequency component) is seen at 50 mmHg of LBPP as compared to baseline. A significant increase in the LF implies that there is a sympathetic predominance in the body during LBPP. Increased sympathetic activity by microgravity simulation by HDT is shown by many other studies [8,9,19]. Thus, it appears that the average heart rate remains the same. The variability (HRV) does not change in the time domain; the frequency domain analysis reveals an increase in the sympathetic drive to heart (LF-HRV) along with the withdrawal of the sympathetic drive to heart (HF-HRV) at 50 mmHg of LBPP.

Garvin et al. (2013) [23] recorded direct recordings of muscle sympathetic nerve activity (MSNA) in subjects with pneumatic anti-shock trousers (PASG) and showed a significant increase in blood pressure by activating a sympathetically mediated reflex by compressing the abdomen but not the leg. PASG has been proposed to exert a mechanical blood pressure-raising effect by increasing venous return and vascular resistance in the lower body [24].

In the present study, the readiness potential was measured during baseline and 50 mmHg of post-LBPP, which shows that there is an increase in the amplitude of C4site activity associated with the process of movement preparation in the bilateral supplementary motor cortex. Similar results have been reported with studies on LBPP treadmill gait training for neurological patients. In other areas, there were no changes seen. No similar observations were made earlier regarding changes in the LBPP associated with readiness potential. However, others using other methods of simulated microgravity have shown improvement [25] in cognitive parameters, similar to our study. However, some studies showed no [26] or impaired cognition after exposure to LBPP [27,28]. An increase in the amplitude of the readiness potential showed synchronous firing of the neurons of supplementary cortex neurons. As the movement of fingers was the only activity done by subjects pre- and post-LBPP, and there is no change in the complexity of movement, there is no change in the latency of readiness potential.

In this study, we found a significant change in the simple and choice reactions upon exposure to 50 mmHg of LBPP. An improvement in the reaction time can be attributed to an increase in the arousal state of the individual due to exposure to 50 mmHg of LBPP [29]. This finding is similar to the finding of Wollseiffen et al. (2019), who also found that microgravity exposure by parabolic flight also showed an improvement in reaction time for complex tasks (mental arithmetic tasks) and a slight (but not significant) decrease in the latency of auditory event-related potential in the oddball paradigm. An improvement in reaction time might be due to an increase in cerebral blood flow due to an increase in upper body fluid shift caused by LPPP exposure. In vivo and in vitro research by Kohn and Ritzmann (2018) demonstrated that microgravity changes neuronal function by decreasing membrane viscosity due to increasing extracellular fluid [30]. They further hypothesized that at the cellular level, this change occurs as a result of a decrease in the open probability of the neuronal membrane ion channel which will decrease the threshold of neuronal firing and make neurons easily excitable [30].

Limitations of the study

The cardiovascular effects of lower body positive pressure should be studied with a gradual change in LBPP pressure. The neurocognitive effects of lower body positive pressure should be studied for a longer period. Sympathetic activation during exposure to lower body positive pressure should be studied by directly recording muscle nerve sympathetic activity. The neurocognitive effects of lower body positive pressure should be studied using EEG-based evoked potential and cognitive tests such stroop task and oddball paradigm. 

## Conclusions

This is the first study to investigate the effect of lower body positive pressure on motor signal processing, reaction time, and cardiovascular parameters. Our findings showed that LBPP can improve motor signal processing and reaction time, but at the same time, it also leads to sympathetic activation during short-term exposure to LBPP. Lower body positive pressure can be used as a model to study the cardiovascular effect of microgravity, as we have shown that pressure 50 of LBPP can lead to a significant change in blood pressure, stroke volume, and cardiac output. Lower body positive pressure of 50 mmHg impacts both simple and choice reactions, indicating a potential impact on decision-making and executive functions.
